# Speciation: genomic sequence data and the biogeography of speciation

**DOI:** 10.1093/nsr/nwac294

**Published:** 2022-12-28

**Authors:** Norman A Johnson

**Affiliations:** Department of Biology, University of Massachusetts–Amherst, USA

Two papers in this issue show how the use of genomic sequence data can illuminate the biogeography of the speciation process. Here, I provide a backdrop to these studies, with a brief history of the use of markers and the biogeography of speciation, and a look toward what these studies mean for speciation studies.

The biogeographic setting greatly affects how the speciation process will proceed. Allopatric speciation—speciation in the absence of gene flow—is easy to explain and has been well established since the modern synthesis of the middle decades of the 20th century [1–3]. Populations separated by geographic barriers evolve independent genetic changes through various evolutionary processes. Some of these changes in one lineage are incompatible with changes in the other; with sufficient incompatible changes, the two populations evolve substantial reproductive incompatibility. If they are brought together, these now-species will either fail to recognize members from the other as suitable mates and/or fail to generate sufficient numbers of viable, fertile hybrid offspring.

More controversial is speciation that arises in the face of gene flow [4]. Theory suggests that such non-allopatric speciation will be more challenging than allopatric speciation for two reasons. First, gene flow brings an antagonism between selection and recombination. For a species to split and start diverging while in the same place usually requires divergent selection operating on different combinations of alleles that work well together; such combinations can be broken up by gene flow and recombination [5,6]. Second, speciation in the same place requires that the two nascent species coexist without one outcompeting the other [7]. This is most likely accomplished by them diverging in their niches and exploiting resources differently. Such resource partitioning will likely lead to selection operating differently on the nascent species, which is likely to further their differentiation. Non-allopatric speciation may be difficult to initiate but the process may become easier after the initial steps. True sympatric speciation (no geographic barriers involved throughout the process) is at an extreme; it is also the most challenging type of speciation [8].

Inferences of patterns from genetic markers has long been applied to the biogeographic nature of speciation. Back in the 1980s, when electrophoretic variants of enzymes (allozymes) were the most feasible way to get a genome-wide view of population differentiation, this method was used to show that *Rhagoletis pomonella* (the apple maggot) collected from apple trees and those collected from hawthorn trees in the same place in Michigan, United States were substantially genetically differentiated [9]. This result was essential to showing that these so-called host races, which started diverging during the middle part of the 19th century, are potential early steps in non-allopatric speciation. Subsequent uses of nuclear DNA markers in that system provided support for a progression of speciation stages in the *R. pomonella* species complex from host races to nearly fully complete species with at least some of the differentiation occurring non-allopatrically [10]. DNA markers also revealed a complexity in the biogeography of this process: some of the genetic variation used in the divergence of the hawthorn and apple host races evolved far away a long time (in Mexico more than a million years ago) [11].

With the increasing availability of DNA sequence data across the genome, we can make further and stronger inferences regarding the biogeography. If gene flow occurred during the speciation process, we should expect to see extensive heterogeneity of the extent of differentiation between the two nascent species across the genome [8,12,13]. Loci involved in the evolving reproductive isolation should show much higher differentiation because the reproductive isolation inhibits introgression at those places in the genome. Linked sites should also show higher differentiation; but as genetic distance increases from those loci contributing to the reproductive isolation, recombination should lead to less differentiation. Thus, genomes of species that diverged due to speciation with gene flow should show islands of differentiation [13]. Caution with this approach should be taken because recombination rates often vary considerably across the genome, not just from chromosomal inversions and other rearrangements but also due to hotspots and coldspots [14]. With this caveat in mind, genomic approaches can provide clues to the biogeographic nature of speciation and potentially reveal candidates for barrier loci–a genetic change that contributes to reproductive isolation.

In this issue, Sun *et al.* [15] examined two closely related fishes, *Gymnocypris eckloni scoliostomus* and *G. eckloni eckloni*, located in Lake Sunmuco, a high-altitude (4100 meters) lake on the Qinghai-Tibet plateau. In addition to differences in head morphology, these fishes also differ in feeding mode: *G. e. scoliostomus* feeds mainly on plankton at the upper water column while *G. e. eckloni* feeds on a variety of items, including small fishes, lower in the water column. These fishes also differ in spawning time, with *G. e. eckloni* spawning a month earlier than *G. e. scoliostomus*.

The genomes of these fishes show many islands of differentiation, solid evidence against their speciation occurring in allopatry. Instead, the pattern strongly suggests substantial gene flow occurred during the speciation process. The nature of these islands of differentiation can provide further information about the biogeography. These observed genomic islands are fairly large with nearly 90% of the total island length was accounted for by 54 large islands that were longer than 100 kb. The presence of such large islands is contrary to results the researchers obtained when they ran simulations assuming that the fishes were speciating in true sympatry. In the sympatric speciation simulations, they recovered numerous, extremely small islands. This discrepancy is strong evidence that the fishes did not speciate under true sympatry. Instead, most likely, the setting for the speciation process was micro-parapatry wherein the evolving fishes had distinct but overlapping ranges on a fine-scale mosaic. Most likely, gene flow was not zero, but it was not complete either, during the initial stages of speciation.

The islands contain a total of 226 genes. Although many of these genes may be incidental to the speciation process, such genes are useful candidates for further analysis especially as many show the signature of selection. Intriguingly, genes involved in olfactory transduction pathways and similar processes are over-represented among those found in the islands of differentiation. This suggests that olfaction phenotypes may have been targets of the divergent selection involved in the speciation process.

A second study by Wang *et al*. [16] in this issue found small, numerous islands of introgression between two species of mangroves: *Rhizophora mucromata* (the loop-root mangrove) and *R. stylosa* (the spotted mangrove). These species, which have overlapping distribution in the Indo-Pacific region, have evolved ecological differences and are generally considered ‘good species’. In regions where the species are found together, the researchers found numerous small introgressed segments. A large proportion of the genome can be introgressed between these apparently good species. This finding, if common, may give support to reconsidering the biological species as currently defined [4,12].

Wang *et al*. [16] also found considerably large regions of the genome without introgressions. Such regions are of interest as well as a lack of introgression would be expected if a barrier locus is nearby. Barrier loci should hinder the local exchange of genes. One intriguing candidate barrier locus found in a region without introgressions is Embryonic flower 1 (*EMF1*); in *Arabidopsis thaliana*, it has manifold effects including changes in the architecture of shoots and the timing of flowers [17].

The researchers might have been fortunate to observe a case of extensive introgression in these mangrove species [16]. Such cases may be rather rare and depend on the timing of the secondary contact. Consider the counterfactuals: secondary contact earlier in the speciation process may have halted or reversed the course of speciation while later secondary contact may have led to far less introgression.

The studies by Sun *et al*. [15] and Wang *et al*. [16] show how genomic data can elucidate the biogeographic setting for non-allopatric studies. These studies and others (reviewed in [8,13]) present a picture of complexity. Likely, most cases of non-allopatric speciation will not be at the other extreme of true sympatry. The studies [15,16] also show the utility of simulation modeling. More theoretical work [8] taking into account these empirical studies is also warranted. Finally, given the heterogeneity of recombination rates across the genome and how this variation can affect studies of islands of differentiation, the genomic studies should strive to examine patterns of recombination, if feasible.
